# Comparative Approaches of Antibiotic Therapy and Surgical Intervention in Managing Recurrent Perirectal Abscess in Children Under Two Years Old

**DOI:** 10.34172/mejdd.2025.417

**Published:** 2025-04-30

**Authors:** Amrollah Salimi, Mohadeseh Najafi, Ahmad Kachoie, Mostafa Vahedian, Enayatollah Noori

**Affiliations:** ^1^Department of Surgery, School of Medicine, Shahid Beheshti Hospital, Qom University of Medical Sciences, Qom, Iran; ^2^Department of Family and Community Medicine, School of Medicine, Spiritual Health Research Center, Qom University of Medical Sciences, Qom, Iran

**Keywords:** Abscess/drug therapy, Anal canal, Child, Hospitalized, Incision-drainage, Recurrence, Spontaneous drainage, Surgery, Perianal abscess, Children, Antibiotics

## Abstract

**Background:**

Recurrent perirectal abscesses in children under 2 years pose management challenges due to high relapse risks, with limited evidence comparing long-term outcomes of antibiotic therapy versus surgical drainage. This study aimed to compare antibiotic treatment approaches and surgical interventions in managing this condition.

**Methods:**

This retrospective cohort study analyzed 336 medical records of children under two years old with perianal abscesses treated at Hazrat Masoumeh Children’s Hospital from 2012-2019. Patients were divided into three groups: antibiotic treatment only, spontaneous drainage, and incision and drainage. Data, including demographics, abscess characteristics, and treatment outcomes (recurrence, fistula formation), were analyzed using SPSS software version 26.

**Results:**

A retrospective cohort study of 336 children with perianal abscesses found no significant differences in age, weight, sex, or nutritional status among the three treatment groups (*P*>0.05). However, statistically significant differences were observed in abscess location (distance from the anus) and size, with the incision and drainage group having larger and more distally located abscesses (*P*<0.05). Colic was less prevalent in the antibiotic-only group. The spontaneous drainage group had a significantly higher recurrence rate (37%). Antibiotic type distribution was similar across groups. The antibiotic-only group showed symptom resolution within 3-10 days, averaging 6 days.

**Conclusion:**

In general, according to the lowest rate of recurrence in the group receiving antibiotics and the average duration of short treatment of 6 days, the antibiotic approach is the preferred and primary method for the treatment and prevention of abscess recurrence.

## Introduction

 Perianal abscess is an anorectal disorder affecting children and adults and causing significant discomfort. It is more common in male patients, with peak onset occurring in those under one year of age.^1-4^ abnormal anal crypts (Morgagni crypts) are believed to be the underlying cause of perianal abscesses in children. Infection in these crypts can result in abscesses, which may heal spontaneously, reappear, or develop into a persistent fistula opening into the rectum.^5,6^ An abscess is an acute infection that penetrates the overlying skin and soft tissues of the anal region via the ducts of the anal glands. The most common treatment for perianal abscess is incision and drainage, typically performed in healthcare settings. A common complication following perianal abscess treatment is the risk of recurrence and the subsequent formation of an anal fistula, estimated to occur in approximately 7 weeks post-initial incision and drainage.^7-11^ Conversely, conservative management is believed to be a safe and effective approach for most perianal abscesses, which is associated with lower recurrence rates and fistula formation. Incision and drainage should be reserved for abscesses showing signs of potential spread or lacking evidence of spontaneous drainage.^12^ While some surgeons favor surgical intervention, others advocate for conservative management. The debate largely centers on surgery’s necessity and optimal timing should conservative treatment fail.^13^ Recurrence or extension of a perianal fistula after treatment of a perianal abscess has been reported in 0% to 85% of cases. This wide range may be attributable to variations in treatment approaches, such as conservative management versus surgical intervention.^14^ The formation of a fistula and recurrence of an abscess after surgical treatment is associated with complications such as prolonged hospital stay, the need for reoperation, patient discomfort, increased medical costs, and a significant economic burden. Numerous theories have been proposed to explain fistula formation following incision and drainage of a perianal abscess. Since infection of the perianal crypts is considered the primary cause of initial abscess formation, one theory regarding fistula formation is the persistence of underlying infection. In cases of fistula development, in addition to the pain caused by the fistula itself, the patient is at a higher risk of recurrent abscess formation. This has led surgeons to consider prophylactic measures to reduce the incidence of postoperative fistula formation, including the initiation of prophylactic antibiotic therapy.^9^ The management and treatment of perianal abscesses and their complications, such as anal fistulas, recurrent infections, etc., remain controversial among pediatric surgeons. Furthermore, this condition and its complications exhibit high prevalence in children, imposing a significant psychological and economic burden on families and society. Various studies have offered differing opinions on the optimal management of this disease and its sequelae.

 This study focuses on managing recurrent perianal abscesses in children under 2 years of age, a population largely neglected in previous research. The study directly compares the efficacy of antibiotic and surgical treatments, evaluating short-term outcomes, long-term effects, and quality of life. Employing a multidisciplinary approach, incorporating the perspectives of various specialists, and considering the influence of local contextual factors on treatment outcomes, this study aimed to investigate and compare the outcomes of surgical and conservative approaches in treating perianal abscesses in children under 2 years of age.

## Materials and Methods

 This was a retrospective cohort study. The study population included children under 2 years of age with perianal abscesses referred to Hazrat Masoumeh Children’s Hospital in Qom from 2012 to 2019. The required sample size in this study was calculated using the following formula and considering a type I error of 5%, a power of 0.8, and a fistula recurrence rate in the medical and surgical groups of 12.7% and 24.6%, respectively. Based on similar studies,^10^ the largest sample size was 167 people in each group, with a total of 336 people included in the study. Sampling was conducted using the convenience method so that the files of 336 children were divided into three groups: Group A (antibiotic treatment only), group B (spontaneous drainage), and group C (incision and drainage). Patients in groups B and C did not receive any antibiotics.

 Inclusion criteria for the study were children under two years of age with perianal abscesses. Exclusion criteria were patients with systemic diseases such as leukemia and inflammatory bowel disease, Hirschsprung’s disease, anorectal malformations, and immunodeficiency. Patients receiving immunosuppressive medications were also excluded. Following the acquisition of ethical approval from the Ethics Committee of Qom University of Medical Sciences (IR.MUQ.REC.1400.226), the researcher began reviewing patient records. Variables extracted from the medical records and subjected to analysis included patient demographics, weight at the time of diagnosis, lesion location, history of constipation, positive family history, treatment methods and antibiotic use, type of antibiotic, abscess recurrence (defined as a repeatedly forming abscess in the same location), development of an anal fistula, symptom presentation, abscess distance from the anal verge, feeding method (breast milk, formula, or a combination), history of colic, and abscess size.

 The cephalexin dose in this study was 50 mg/kg daily, and co-amoxiclav was 30 mg/kg daily in divided doses.

 The follow-up period of the patients was also carried out from 2 months to 2 years.

 Data from 336 children’s medical records were entered into SPSS software version 26. The normality of the data was assessed using the Kolmogorov-Smirnov test. Chi-square tests were used to analyze categorical data, while t-tests and ANOVA were employed for continuous data analysis. The significance level was set at α = 0.05 for all tests.

## Results

 The age, weight, and sex distributions among the groups show no statistically significant differences, with patients having a mean age of approximately 2.2 years and a weight of around 4.3 kg. The sex ratio is predominantly male across all groups, with no significant variance. Nutritional status reflects a majority of patients receiving breast milk, with similar proportions in each treatment group, suggesting consistent nutritional support. The slight variations in formula feeding and severe nutritional status among the groups also lack statistical significance (Table 1).

**Table 1:** Demographic and nutritional variables in pediatric patients undergoing different treatments for perirectal abscesses

		**Group**			***P*** ** value**
		**Antibiotic therapy (n=66)**	**Spontaneous discharge (n=54)**	**Incision and drainage (n=216)**	
Age		2.39 ± 1.4	2.02 ± 1.2	2.36 ± 1.6	0.331
Weight		4.40 ± 0.91	4.20 ± 0.92	4.35 ± 1.13	0.566
Sex	Boy	61 (92.4 %)	50 (92.6 %)	205 (94.9 %)	0.671
	Girl	5 (7.6 %)	4 (7.4 %)	11 (5.1 %)	
Nutrition	Breast milk	52 (78.8 %)	41 (75.9 %)	157 (72.7 %)	0.831
	Formula	5 (7.6 %)	5 (9.3 %)	26 (12 %)	
	Severe malnutrition	9 (13.6 %)	8 (14.8 %)	33 (15.3 %)	

Table 2 shows that there was a statistically significant difference (*P* < 0.05) in abscess distance from the anus and abscess diameter between the groups. The group treated with antibiotics and spontaneous drainage had a smaller distance from the anus and a smaller diameter compared with the incision and drainage group. Colic severity also differed significantly between groups (*P* = 0.021), with a lower percentage of patients in the antibiotic-only group experiencing colic. The abscess recurrence rate was higher in the spontaneous drainage group (37%), and this difference was statistically significant (*P* = 0.022). Finally, the distribution of antibiotic type used was comparable between groups and showed no statistically significant difference (*P* = 0.819). Furthermore, the minimum duration of symptom resolution in the antibiotic group was 3 days after the start of treatment, and the maximum duration was 10 days. The average duration of response to treatment was 6 days.

**Table 2:** Clinical outcomes of antibiotic therapy, spontaneous discharge, and incision and drainage in the management of perirectal abscesses in pediatric patients

		**Group**			***P*** ** value **
		**Antibiotic therapy (n=66)**	**Spontaneous discharge (n=54)**	**Incision and drainage (n=216)**	
Abscess distance from anal		17.47 ± 1.5	17.67 ± 1.8	18.19 ± 1.6	0.002
Abscess diameter (mm)		6.91 ± 1.14	7.56 ± 1.17	7.65 ± 1.12	0.001
Constipation	Yes	9 (15.3 %)	9 (18.8 %)	47 (24.2 %)	0.298
	No	50 (84.7 %)	39 (81.3 %)	147 (75.8 %)	
Colic Severity	None	21 (31.8)	9 (16.7 %)	81 (37.7 %)	0.021
	Mild	22 (33.3 %)	24 (44.4 %)	81 (37.7 %)	
	Severe	23 (34.8 %)	21 (38.9 %)	53 (24.7 %)	
Recurrence	Yes	11 (16.7 %)	20 (37 %)	47 (21.8 %)	0.022
	No	55 (83.3 %)	34 (63 %)	169 (78.2 %)	
Antibiotic	Cephalexin	49 (75.4 %)	41 (80.4 %)	162 (77.1 %)	0.819
	Co-amoxiclav	16 (24.6 %)	10 (19.6%)	48 (22.9 %)	

## Discussion

 This study shows a mean age of approximately 32.2 ± 1.57 months for children with abscesses. This finding suggests the possibility of abscess onset within this age range and could be considered as one of the potential causes of unexplained irritability in children of this age group. Previous studies, such as the research by Gong and colleagues, reported the highest incidence of abscesses at the age of 2 months.^6^ Similar results were observed in other studies, all pointing to a higher prevalence of abscesses in infancy.^1,8^ The mean weight among the group of children affected was 34.4 kg. According to the weight-for-age percentiles chart, this average weight corresponds to the 2.5th percentile for boys and the 10.2nd percentile for girls, based on the average age. Therefore, the results indicate that children who fall into lower percentiles for weight relative to their age are more likely to develop perianal abscesses. It is recommended that this relationship be investigated more thoroughly in future studies. In the 2017 study by Ghahramani and colleagues^9^ on adults, the relationship between body mass index (BMI) and the odds of fistula formation was investigated, which was non-significant. Furthermore, the association between weight and treatment choice in similar pediatric studies reviewed in our previous work has not been explored. Furthermore, the antibiotic, incision, and drainage treatment groups exhibited approximately similar weight-for-age percentiles near the overall mean in both sexes. Only the spontaneous drainage group showed a significantly lower weight-for-age percentile than the mean in both sexes. Despite the lack of a statistically significant association between sex and treatment group in children with abscesses, this study, like others, found that male patients constituted the majority (94%) of the total number of children with abscesses.^1,6,8^ A significant correlation was observed between colic severity and the treatment group in children with abscesses. This suggests spontaneous resolution is less likely in infants with a history of severe colic. Consequently, in the majority of children with a history of severe neonatal colic, after a course of antibiotic therapy and in the absence of improvement, the likelihood of spontaneous abscess drainage is reduced, increasing the probability of requiring surgical intervention (incision and drainage). Conversely, children with a history of severe colic demonstrated a lower likelihood of requiring incision and drainage. In other words, in this cohort, antibiotic therapy and spontaneous resolution were more successful following a course of antibiotics, resulting in a reduced need for surgical intervention. Previous studies have not investigated this variable’s correlation with treatment modality and recurrence. A statistically significant correlation was observed between the mean abscess diameter and the treatment group. The mean abscess diameter was the smallest in the antibiotic group, followed by the spontaneous drainage and incision and drainage groups. Therefore, it appears that antibiotic therapy yielded the best results in the group with the smallest abscess diameter, and as the abscess diameter increased, the treatment approach shifted towards intervention and incision and drainage. This aligns with the findings of Boenicke et al^13^ who reported a direct correlation between abscess size and recurrence rate. A statistically significant correlation was observed between the mean anal-rectal distance and the treatment group. The antibiotic group exhibited the shortest mean anal-rectal distance, while the incision and drainage group showed the longest. Therefore, as the anal-rectal distance increases, the treatment approach shifts from antibiotic therapy to incision and drainage. Previous studies reviewed in this analysis did not investigate the correlation between this variable and the treatment group, nor the risk of recurrence. A statistically significant difference in abscess recurrence rate was observed among treatment groups. The antibiotic treatment group demonstrated the lowest recurrence rate, while the group with spontaneous drainage and debridement showed the highest. In contrast to our findings, the studies by Boenicke et al,^13^ Juth Karlsson et al,^5^ and Ezer et al^8^ reported no significant difference in recurrence rates between conservative and surgical treatment approaches. In contrast, the 2017 study by Gong et al^6^ found that conservative treatment with topical antibiotics (polymyxin) resulted in a lower recurrence rate compared with surgical treatment. Furthermore, the study by Afsarlar and colleagues^15^ demonstrated that postoperative use of penicillin and cephalosporins resulted in a lower incidence of recurrence and fistula formation compared with the group that did not receive antibiotics. A 2017 study by Ghahramani et al^9^ found that postoperative treatment with ciprofloxacin and metronidazole decreased the likelihood of fistula formation in adults with perianal abscesses.

 The limitations of this study include a small sample size and a cross-sectional design, which may affect the generalizability of the results. Additionally, there is a need for greater attention to individual variables and treatment variability. These factors are significant and should be more thoroughly investigated in future research to enhance the credibility and accuracy of the findings. The study’s findings suggest a correlation between abscess size and proximity to the anal verge and the likelihood of successful antibiotic-only treatment in children, resulting in a lower recurrence rate. Smaller abscesses located closer to the anal verge demonstrated a higher probability of resolving with antibiotics alone, thus minimizing the need for surgical intervention and reducing the risk of recurrence.

## Conclusion

 This study investigated several factors, including abscess recurrence, antibiotic type, treatment duration, abscess diameter, and its distance from the anal verge. The results indicate that, given the lowest recurrence rate and a short average treatment duration of 6 days (maximum 10 days), an antibiotic approach is the preferred initial method for treatment and recurrence prevention. Furthermore, the group treated with drainage under sterile conditions showed a lower recurrence rate than those with spontaneous abscess drainage. The type of antibiotic initially used did not significantly impact abscess recurrence.
